# The probability of readmission within 30 days of hospital discharge is positively associated with inpatient bed occupancy at discharge – a retrospective cohort study

**DOI:** 10.1186/s12873-015-0067-9

**Published:** 2015-12-14

**Authors:** Mathias C. Blom, Karin Erwander, Lars Gustafsson, Mona Landin-Olsson, Fredrik Jonsson, Kjell Ivarsson

**Affiliations:** Department of Clinical Sciences Lund, Lund University, HS 32, EA-blocket, 2nd floor, SE-22185 Lund, Sweden; IK-enheten, Helsingborg general hospital, S Vallgatan 5, SE-25187 Helsingborg, Sweden; Department of Pre- and intrahospital Emergency Medicine, Helsingborg general hospital, S Vallgatan 5, SE-25187 Helsingborg, Sweden

**Keywords:** Emergency medicine, Bed occupancy rate, Hospital readmission, Premature hospital discharge

## Abstract

**Background:**

Previous work has suggested that given a hospital’s need to admit more patients from the emergency department (ED), high inpatient bed occupancy may encourage premature hospital discharges that favor the hospital’s need for beds over patients’ medical interests. We argue that the effects of such action would be measurable as a greater proportion of unplanned hospital readmissions among patients discharged when the hospital was full than when not. In response, the present study tested this hypothesis by investigating the association between inpatient bed occupancy at the time of hospital discharge and the 30-day readmission rate.

**Methods:**

The sample included all inpatient admissions from the ED at a 420-bed emergency hospital in southern Sweden during 2011–2012 that resulted in discharge before 1 December 2012. The share of unplanned readmissions within 30 days was computed for levels of inpatient bed occupancy of <95 %, 95–100 %, 100–105 % and >105 % at the hour of discharge. A binary logistic regression model was constructed to adjust for age, time of discharge, and other factors that could affect the outcome.

**Results:**

In all, 32,811 visits were included in the study, 9.9 % of which resulted in an unplanned readmission within 30 days of discharge. The proportion of readmissions was 9.0 % for occupancy levels of <95 % at the patient’s discharge, 10.2 % for 95–100 % occupancy, 10.8 % for 100–105 % occupancy, and 10.5 % for >105 % occupancy (*p* = 0.0001). Results from the multivariate models show that the OR (95 % CI) of readmission was 1.11 (1.01–1.22) for patients discharged at 95–100 % occupancy, 1.17 (1.06–1.29) at 100–105 % occupancy, and 1.15 (0.99–1.34) at >105 % occupancy.

**Conclusions:**

Results indicate that patients discharged from inpatient wards at times of high inpatient bed occupancy experience an increased risk of unplanned readmission within 30 days of discharge.

## Background

Two previous studies have shown that inpatient bed occupancy at the time of patient presentation in the emergency department (ED) was negatively associated with the probability of hospital admission, yet not the probability of an unplanned 72-h revisit to the ED [1, 2].

As a topic, the availability of inpatient beds has attracted much interest from a systems perspective, from which several models suggest an association between average bed occupancy and frequency of acute bed shortage [3–6]. General principles of queuing theory maintain that variation in the number of hospital admissions and inpatient length of stay (IPLOS) explains most of the variation in bed occupancy [7–11], though others argue that the former explains more variation than the latter [12].

Different strategies have been proposed for anticipating and accommodating periods of acute bed shortage. Several studies have suggested that reducing the variability in the volume of planned hospital admissions will smoothen inpatient bed occupancy and thereby reduce the frequency of acute bed shortages [10, 13, 14]. Others have suggested that pooling resources throughout large hospital systems might promote higher average bed occupancy rates than seen in smaller hospital systems, yet without acute bed shortages [5, 8]. At the same time, scheduling discharges from inpatient wards earlier in the day has been thought to decrease conflicting demand for inpatient beds between patients not yet discharged and patients waiting for admission, especially since most admissions from an emergency department occur in the afternoon [15, 16]. This last idea is particularly interesting, since frequent shortages of open inpatient beds have been attributed with triggering a mechanism by which the demand for accommodating new admissions drives hospital discharge, thereby leaving patients at risk of premature discharge [8]. If so, then such effects should appear as a positive association between inpatient bed occupancy at time of discharge and rate of unscheduled hospital readmissions within 30 days.

The aim of the present study was to test this hypothesis by investigating the association between inpatient bed occupancy at the time of discharge from an inpatient ward and the probability of an unplanned hospital readmission from the ED within 30 days. This study is exploratory and seeks to develop hypotheses for further research in the field.

## Methods

### Study design

In this retrospective cohort study, the sample included all episodes of inpatient care experienced by patients admitted from the ED to the inpatient setting at a 420-bed hospital in southern Sweden during 2011–2012 and discharged before 1 December 2012. The 30-day readmission rate for discharged patients at this hospital was previously estimated to be about 9 %. For the purposes of this study, an increase of 2 % was considered to be clinically relevant.

To limit bias, the study material was not subject to further restrictions. *Post hoc* power calculations were performed to determine the number of strata (see cut-offs in the Statistical Analysis section) of inpatient bed occupancy to use for group comparisons (α = 0.05, 1-β = 0.80) [17].

### Data sources

Data on inpatient care episodes were retrieved from the hospital billing system PASiS®. Data on hospital occupancy per hour were retrieved from an occupancy database used by hospital management for purposes of quality assurance. Data on ED visits were retrieved from the ED information system Patientliggaren®. Data gathering and linking were performed by the hospital informatics unit using QlikView® software. The head of the division (KI) and the chair of the ED (FJ) granted access to data.

### Setting

Helsingborg general Hospital is one of four hospitals that provide emergency care in the region of southern Sweden called Region Skåne. Its ED serves a population of roughly 250,000, which expands to more than 300,000 in summer due to tourism. The hospital is a teaching hospital that offers education for medical students as well as emergency medicine residents.

Its ED is separated into units by specialty, and in 2010, a complementary unit staffed by emergency physicians capable of handling all but psychiatric, otolaryngologic, ophthalmologic, and pediatric complaints was introduced that currently operates from 8:00–23:00 daily. There are separate EDs for children (<18 years of age) with medical conditions and for patients with obstetric, gynecologic, psychiatric, or ophthalmologic complaints. Patients admitted from these EDs were excluded from the study. Patients with suspected hip fractures or ST-elevation Myocardial Infarction (STEMI) diagnosed in an ambulance bypass the ED and were thus also excluded. Hand surgery, neurosurgery, and thoracic surgery are not available at the hospital, and the availability of endovascular surgery and PCI are limited after hours (17:00–08:00). Patients with such needs are referred to Skåne University Hospital (SUS) and were thus also excluded. At times of pronounced bed shortage, some patients are admitted from the ED to two other hospitals in the region. Patients admitted to these hospitals at index were likewise excluded.

### Statistical analysis

Unplanned readmissions were defined as readmissions to the hospital through the ED within 30 days of discharge. We computed the readmission rate for inpatient bed occupancy rates of <95 %, 95–100 %, 100–105 %, and >105 % and compared proportions using Fisher’s exact test. Inpatient bed occupancy <85 % has traditionally been used for the reference level in the field, following Bagust et al [4]. Since the mean bed occupancy at the study site is around 95 %, <85 % is likely to reflect an artificial situation and hence <95 % was selected for reference. *Post hoc* power analysis revealed that the power to detect the pre-specified difference (2 %) was 84.2 % for the smallest category (>105 %). A binary logistic regression model was constructed in order to adjust for confounders and other factors liable to affect the outcome. Variables considered for inclusion in the model were sex, age group, IPLOS, the admitting specialty at index admission, day of the week of discharge, time of day of discharge (00:00-07:59, 08:00-15:59, 16:00-23:59), and inpatient bed occupancy at discharge. Age was grouped into intervals of 0–18 years, 18–40 years, 40–65 years, and ≥65 years. In Sweden, 18 years is the age of legal adulthood and 65 the age of retirement. For the binary logistic regression models, inpatient bed occupancy was categorized by the same intervals as in the crude analysis.

Predictors were tested for crude association with the outcome before entering the preliminary primary effects model. Associations weaker than *p* = 0.25 but of clinical importance were still included [18]. Multicollinearity testing was performed using Spearman correlation [19], and the selection of interaction terms screened for inclusion in the final models was governed by perceived clinical significance determined *a priori*. Variables were manually added to the models. Model fit was evaluated using Nagelkerke’s *R*^*2*^*,* the Hosmer & Lemeshow test and ROC-curves. The association between each predictor and the outcome was addressed by the -2LL and Wald statistic. Models were screened for influential cases by addressing standardized residuals and Cook’s distance. To prevent overfitting, the final model selected was that with the highest explanatory value relative to the number of predictors [19]. Statistical analyses were performed with IBM® SPSS® version 22. Data was anonymized before analysis. The regional ethical review board in Lund granted ethical approval for the study (dnr 2013/11).

## Results

**Fig. 1:**
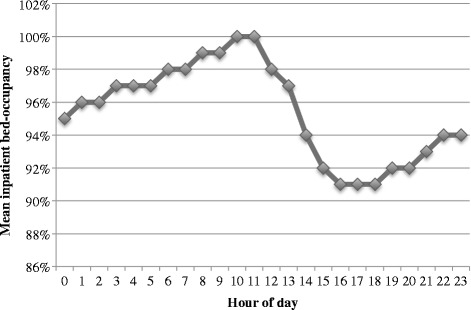
Mean inpatient bed occupancy per hour during the study period

In sum, 160,462 visits to the main ED and the separate EDs for children with medical conditions (<18 years of age) and patients with ophthalmologic complaints, were registered in Patientliggaren® during 2011–2012. Of these, 39,095 resulted in admission, of which 1,444 (3.7 %) lacked a corresponding inpatient episode in PASiS® and were therefore excluded. A further 2,388 (6.1 %) were excluded because they were not admitted through the main ED at index, as were another 723 (1.8 %) for being transferred to other hospitals during their index inpatient episode. Lastly, another 1,729 (4.4 %) were excluded because they were discharged from the inpatient setting after 30 November 2012. A total of 32,811 visits were included in the final study, 3,247 (9.9 %) of which resulted in an unplanned 30-day readmission. Average hourly inpatient bed occupancy during the study period is shown in Fig. 1.

### Crude analysis

The proportion of 30-day readmissions after discharge from the hospital when at <95 % occupancy was 9.0 %. If 95–100 % of hospital beds were occupied at the time of discharge, then the readmission rate was 10.2 %. It was 10.8 % for 100–105 % occupancy, and 10.5 % for >105 % occupancy (p = 0.0001). Study power was >85 % for the detected differences, except for when comparing the smallest subgroup (occupancy >105 %) to the reference group, where it was only 62 %. 609/32,811 = 1.9 % of cases were discharged during nighttime (00:00-07:59), 5,477/32,811 = 16.7 % in the afternoon (16:00-23.59) and 26,725/32,811 = 81.5 % during daytime (08:00-15:59).

### Adjusted analysis

**Fig. 2:**
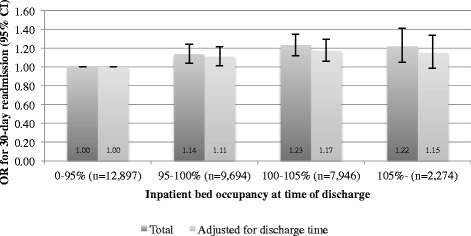
Odds-ratio of 30-day readmission at different levels of inpatient bed occupancy at discharge

All predictors screened for inclusion in the multivariate models were included in the preliminary primary effects models. The final models included occupancy, age group, and specialty unit responsible for admitting the patient at index. A sensitivity analysis was performed that included the effects of the time of day of discharge. Model results agreed with those of the crude analysis and showed that the OR of readmission was 1.14 (95 % CI 1.04–1.24) for patients discharged at 95–100 % occupancy, 1.23 (1.12–1.35) at 100–105 % occupancy, and 1.22 (1.05–1.41) at >105 % occupancy, relative to that of patients discharged at occupancy <95 %. Corresponding numbers for the sensitivity analysis were OR 1.11 (1.01–1.22), 1.17 (1.06–1.29), and 1.15 (0.99–1.34), respectively. See Fig. 2 and Table 1 for a full account of the final model and sensitivity analysis.

**Table 1:** Results from unadjusted and adjusted analysis

	Occ. at discharge	30-day readm.	Reg. coeff.	SE	Wald	*p*	OR	95 % CI low	95 % CI high
Main analysis Nagelkerke's *R* ^*2*^ = 0.030	<95 % (ref) *N* = 12,897	1162 (9.0 %)			21.41	<0.001	ref		
	95–100 % *N* = 9,694	989 (10.2 %)	0.13	0.05	7.76	0.01	1.14	1.04	1.24
	100–105 % *N* = 7,946	857 (10.8 %)	0.21	0.05	18.69	<0.001	1.23	1.12	1.35
	>105 % *N* = 2,274	239 (10.5 %)	0.20	0.08	6.81	0.01	1.22	1.05	1.41
Adjusted for time of discharge Nagelkerke's *R* ^*2*^ = 0.033	<95 % (ref) *N* = 12,897	1162 (9.0 %)			11.49	0.01	ref		
	95–100 % *N* = 9,694	989 (10.2 %)	0.11	0.05	5.19	0.02	1.11	1.01	1.22
	100–105 % *N* = 7,946	857 (10.8 %)	0.16	0.05	10.15	0.001	1.17	1.06	1.29
	>105 % *N* = 2,274	239 (10.5 %)	0.14	0.08	3.31	0.07	1.15	0.99	1.34

**Fig. 3:**
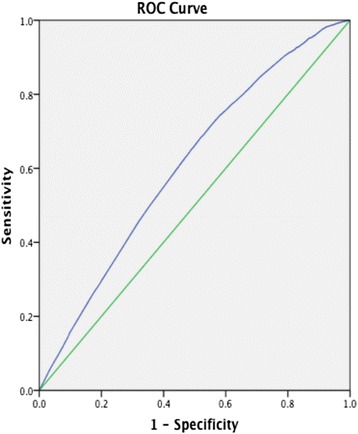
ROC-curve from main analysis. AUC 0.61 (95 % CI 0.60-0.62)

**Fig. 4:**
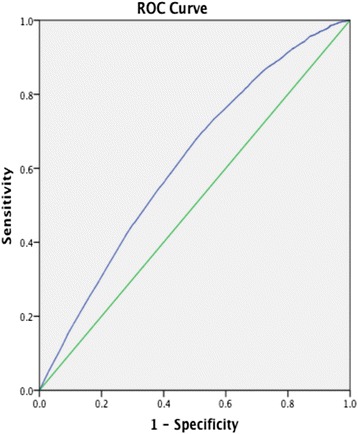
ROC-curve from sensitivity analysis. AUC 0.62 (95 % CI 0.61-0.63)

The insignificant results of the Hosmer & Lemeshow test (*p* = 0.505 and *p* = 0.707 for the main analysis and the sensitivity analysis, respectively) suggested that the models fitted the data well. Nagelkerke's *R*^*2*^ indicated that the models were of fairly low explanatory value, supported by the presence of some large residuals. The area under the ROC curve (AUC) was 0.61 (95 % CI 0.60-0.62) for the main analysis and 0.62 (95 % CI 0.61-0.63) for the sensitivity analysis, suggesting limited to moderate explanatory value (see Figs. 3 and 4).

## Discussion

Both the crude and adjusted results suggest a positive association between inpatient bed occupancy at the hour of patient discharge and the 30-day readmission rate. Even though the differences were smaller than what was considered clinically meaningful prior to conducting the study, the *post hoc* power calculation revealed adequate statistical power (>85 %) for the detected differences between each occupancy category and the reference, except for the smallest subgroup (cases discharged at occupancy >105 %). We argue that these findings support the hypothesis that high inpatient bed occupancy is associated with premature hospital discharges. The most notable absolute difference is between either of the subgroups with cases discharged at high inpatient bed occupancy and the reference category (occupancy <95 %), rather than between those subgroups themselves. This suggests that the mechanism causing premature discharges becomes effective when the hospital is close to full (i.e. >95 % occupied). The results were attenuated when time of discharge was adjusted for in the sensitivity analysis. It is hard to say whether the time of discharge or the occupancy at discharge is the major driver behind the 30-day readmission rate, since it is possible that high inpatient bed occupancy causes delays in discharges (higher census causes longer discharge rounds), so that some of the effect attributed to time of discharge in the sensitivity analysis is really mediated by the inpatient bed occupancy rate. Whichever the case, inpatient bed occupancy remained a significant predictor of 30-day readmissions in the sensitivity analysis for all but the smallest subgroup (cases discharged at occupancy >105 %). This is likely to reflect the small size of this subgroup, which should be collapsed in future studies.

### Limitations

A limitation of the study was that readmissions through other EDs in the region were not detected by the present study design, though hospital management and clinical staff claim that this fraction is small. Some bias may also have been introduced since no cases from December 2012 were included; however, most of the winter season was included and the study period covered 2 years, which would limit the effects of such bias to some extent. Moreover, even though the 30-day readmission rate is frequently used as a quality measure [20–22], it is too blunt to capture all aspects of quality of care. We chose the 30-day readmission rate as the outcome measure since it has been studied before and is considered to reflect various insufficiencies in a healthcare system. Several patient factors [20], inter-hospital variation [21, 22], and specific interventions aimed at reducing hospital readmissions [23–25] have been suggested to affect readmission rates. Many of these were not adjusted for (e.g. diagnosis, co-morbidity, and occupational status), since they are unfortunately unavailable from the data sources available to us. The limited predictive ability of the multivariable models is also implied by the fairly low areas under the ROC-curves and the values of the Nagelkerke's *R*^*2*^ coefficients. Despite this, we view the agreement between crude-, multivariable and sensitivity analyses as a relevant signal in the data not to be neglected. Moreover, some of the effect may reflect the undifferentiated status of the study population, suggesting that future studies should aim at describing the effect for limited groups of patients.

## Conclusions

Study results indicate a positive association between inpatient bed occupancy at discharge from inpatient wards and the 30-day readmission rate. Though the prematurity of hospital discharges may not be measurable by a single outcome measure, our results provide support for the hypothesis that high inpatient bed occupancy is associated with premature discharges from inpatient wards and points to the need of studying the subject closer.
